# Tangshen Formula Attenuates Diabetic Nephropathy by Promoting ABCA1-Mediated Renal Cholesterol Efflux in db/db Mice

**DOI:** 10.3389/fphys.2018.00343

**Published:** 2018-04-06

**Authors:** Peng Liu, Liang Peng, Haojun Zhang, Patrick Ming-Kuen Tang, Tingting Zhao, Meihua Yan, Hailing Zhao, Xiaoru Huang, Huiyao Lan, Ping Li

**Affiliations:** ^1^Beijing Key Lab Immune-Mediated Inflammatory Diseases, Institute of Clinical Medical Sciences, China-Japan Friendship Hospital, Beijing, China; ^2^Graduate School of Peking Union Medical College, Chinese Academy of Medical Science & Peking Union Medical College, Beijing, China; ^3^Li Ka Shing Institute of Health Sciences and Department of Medicine and Therapeutics, The Chinese University of Hong Kong, Hong Kong, Hong Kong; ^4^Department of Anatomical and Cellular Pathology, The Chinese University of Hong Kong, Hong Kong, Hong Kong

**Keywords:** Tangshen formula (TSF), diabetic nephropathy (DN), renal cholesterol efflux, ABCA1, Abca1-SiRNA

## Abstract

The commonly prescribed Tangshen Formula (TSF) is a traditional Chinese formulation that has been shown to reduce plasma lipid metabolism and proteinuria and improve the estimated glomerular filtration rate (eGFR) in patients with diabetic kidney disease. This study investigated the underlying mechanism whereby TSF regulates renal lipid accumulation and ameliorates diabetic renal injuries in spontaneous diabetic db/db mice and *in vitro* in sodium palmitate (PA)-stimulated and Abca1-SiRNA-transfected mouse tubular epithelial cells (mTECs). The results revealed that TSF treatment significantly ameliorated the renal injuries by lowering urinary albumin excretion and improving renal tissue injuries in diabetic (db/db) mice. Interestingly, the treatment with TSF also resulted in decreased cholesterol levels in the renal tissues of db/db mice, which was associated with increased expression of the peroxisome proliferator-activated receptor γ coactivator 1-α (PGC-1α), the Liver X receptors (LXR), and ATP-binding cassette subfamily A member 1 (ABCA1), suggesting that TSF might attenuate diabetic kidney injury via a mechanism associated with improving cholesterol efflux in the diabetic kidney. This was investigated *in vitro* in mTECs, and the results showed that TSF reduced the PA-stimulated cholesterol accumulation in mTECs. Mechanistically, the addition of TSF was capable of reversing PA-induced downregulation of PGC-1α, LXR, and ABCA1 expression and cholesterol accumulation in mTECs, suggesting that TSF might act the protection via the PGC-1α-LXR-ABCA1 pathway to improve the cholesterol efflux in the renal tissues of db/db mice. This was further confirmed by silencing ABCA1 to block the promotive effect of TSF on cholesterol efflux *in vitro*. In conclusion, TSF might ameliorate diabetic kidney injuries by promoting ABCA1-mediated renal cholesterol efflux.

## Introduction

Diabetic nephropathy (DN) is one of the major long-term microvascular complications of type 2 diabetes mellitus (T2DM) and is a leading cause of end-stage renal disease (ESRD) worldwide (Perco and Mayer, 2018). Hyperlipidemia, such as hypercholesterolemia, has been proposed as a risk factor for initiation and progression of DN (Kim et al., 2018). In patients with DN and in animal models, cholesterol accumulation in the kidney accelerated glomerulosclerosis and interstitial fibrosis by inducing oxidative stress and inflammatory response (Ruan et al., 2009; Kim et al., 2018). Renal cholesterol accumulation is a characteristic of DN (Pedigo et al., 2016), which occurs together with alterations in cholesterol synthesis, cholesterol efflux and cholesterol uptake (Declèves et al., 2014). Recent studies suggested that the decrease of cholesterol efflux is a risk factor for cholesterol accumulation (Merschergomez et al., 2013).

The ATP-binding cassette (ABC) transporters mainly mediate the cellular cholesterol efflux pathway, among which ABCA1 is the most prominent member (Schumacher and Benndorf, 2017). ABCA1 plays a key role in promoting the efflux of cellular cholesterol to apolipoprotein A1 (apoA1; Wang et al., 2000). In patients with T2DM and incipient or overt nephropathy, the capacity of ABCA1-mediated cholesterol efflux in macrophages and podocytes is impaired (Zhou et al., 2008; Pedigo et al., 2016). Additionally, a recent study indicated that the increased ABCA1-mediated cholesterol efflux attenuated renal cholesterol accumulation in DN patients (Ganda et al., 2017).

Although considerable progress has been made in elucidating the molecular mechanisms involved in DN, adequate treatment options for this disease remain limited (Marathe et al., 2017). Patients with diabetic kidney diseases in China have widely received the therapy of Chinese herbal medicine (CHM) (Sun et al., 2016). Tangshen Formula (TSF) is a CHM that is used for treating diabetic kidney diseases, and it significantly reduced proteinuria and improved the estimated glomerular filtration rate (eGFR) among diabetic kidney diseases patients, as demonstrated by a multicenter double-blind randomized placebo-controlled trial (Li et al., 2015). It was demonstrated that TSF decreased plasma lipid metabolism in patients with diabetic kidney diseases (Yu et al., 2011) and reduced hepatic steatosis by inhibiting lipogenesis and increasing fatty acid oxidation in db/db mice (Kong et al., 2016). However, whether TSF decreases renal cholesterol accumulation in diabetes conditions is unclear. In the present study, we found that TSF inhibited renal cholesterol accumulation by promoting ABCA1-mediated cholesterol efflux to ameliorate diabetic kidney injuries in db/db mice.

## Materials and methods

### Herbal formulation and components

TSF granules contain seven natural herbs. The analysis of its composition was performed as previously described (Kong et al., 2016). We prepared and standardized the herbs at Jiangyin Tianjiang Pharmaceutical Co. (Jiangyin, Jiangsu, China). The preparation of the herbal drugs was authenticated and standardized according to the established guidelines in the Chinese Pharmacopoeia 2010. TSF granules for use in the animal experiments were dissolved in distilled water (0.18 g/mL).

### Animals and experimental design

Eight-week-old male C57BLKS/J db/db (*n* = 18) and db/m (*n* = 9) mice were purchased from the Peking University Laboratory Animal Center (Beijing, China). Mice were housed under controlled temperature (23 ± 3°C) and humidity (55 ± 15%), on a 12-h light-dark cycle, and were allowed access to standard food and water *ad libitum*. The db/db mice were divided into two groups (*n* = 9 for each group): one group received TSF by intra-gastric gavage (db/db+TSF, 2.4 g/kg/day) and the other group was administered saline (db/db). The db/m mice were used as controls (db/m). After feeding for 2 weeks, the mice were treated with TSF for 12 weeks and then blood and tissues were collected for further analysis.

The study protocol was approved by the Ethics Committee of the China-Japan Friendship Institute of Clinical Medical Sciences (approval no. 13005). Experiments were performed in accordance with the National Institutes of Health Guiding Principles for the Care and Use of Laboratory Animals.

### Preparation of oleic acid (OA) and sodium palmitate (PA)

Oleic acid (O1008) and sodium palmitate (P9767) were purchased from Sigma-Aldrich (Saint Louis, MO, USA). A 100 mM oleic acid (OA) stock solution was prepared in 0.1 M NaOH by heating at 70°C in a shaking water bath (Cousin et al., 2001). In an adjacent water bath at 55°C, the 100 mM OA stock solution was dissolved at 5 mM in culture medium containing 1% bovine serum albumin. Sodium palmitate (PA) was dissolved at 100 mM in distilled water, shaken at 70°C, dissolved at 5 mM in culture medium containing 1% bovine serum albumin, and then shaken at 37°C. Both solutions of OA and PA were stored at 4°C. The final concentration of OA- and PA-induced cells was 50 μM, and the medium contained 0.01% BSA.

### Cell culture

The mouse tubular epithelial cells (mTECs, a gift from Dr. Jeffrey B. Kopp, NIH, Bethesda, MD, USA) were cultured in a humidified incubator with 5% CO_2_ at 37°C, in DMEM Low Glucose medium (Life Technologies, Gaithersburg, MD, USA), supplemented with 10% FBS (Gibco, Grand Island, NY, USA) as previously described (Zhou et al., 2015). The cells were divided into the following groups: the OA group and the PA group, which were grown with 50 μM oleic acid and 50 μM sodium palmitate, respectively. The PA+TSF 250 and PA+TSF 500 groups were treated with 50 μM sodium palmitate + 250 μg/mL TSF and 50 μM sodium palmitate + 500 μg/mL TSF, respectively (Zhao et al., 2017).

### Cell viability

The MTT [3-(4,5-dimethylthiazol-2-yl)-2,5-diphenyltetrazolium bromide] assay was used to determine the effect of TSF on cell viability. After being fasted, mTECs growing in 96-well plates were incubated with TSF at dosages of 62.5, 125, 250, 500, 1,000, and 2,000 μg/mL for 24 or 48 h. Subsequently, 20 μL of MTT (5 mg/mL) was added to each well, and the cells were further incubated for an additional 4 h. The supernatant was removed, and the formazan crystals were dissolved with 100 μL/well of DMSO, and then shaken 10 min. The optical density was measured at 490 nm using a microplate reader (BioTek, Winooski, VT, USA).

### Transfection of siRNA

The mTECs were transfected with siRNA (20 μM) targeting ABCA1 (ABCA1-SiRNA) or scrambled siRNA as a negative control (Co-ABCA1-SiRNA) using Lipofectamine 3000 reagent (Invitrogen, Carlsbad, CA, USA) according to the manufacturer's instructions. The siRNA sequences were as follows: sense, 5′-CCAGCUGAAGGGCUGGAAATT-3′ and antisense, 5′-UUUCCAGCCCUUC AGCUGGTT-3′ (purchased from GenePharma, Shanghai, China). After siRNA transfection, cells were incubated with or without TSF for 24 h (Meng et al., 2015). Then cells were incubated with OA or PA.

### Measurement of serum and urinary parameters

The mice were kept in metabolic cages (Fengshi Inc., Suzhou, JS, China) and 24 h urine samples were collected every 4 weeks for urine volume and urine albumin-to-creatinine ratio (UACR) measurement. All the mice were fasted overnight before collection of blood samples and euthanasia. Serum triglycerides (TG), total cholesterol (TC), low-density lipid cholesterol (LDL-C), high-density lipid cholesterol (HDL-C), and urine creatinine were measured using an automatic analyzer (Abbott Diagnostics, Abbott Park, IL, USA). Urine albumin was measured by enzyme linked immunosorbent assay (ELISA) using an ELISA Quantitation Set kit (Bethyl Laboratories Inc., Montgomery, TX), according to the manufacturer's instructions.

### Measurement of cholesterol levels

Cholesterol levels were quantified in the kidneys of the mice and mTECs by colorimetric assay kit (Total Cholesterol Colorimetric Assay kit, Cell Biolabs Inc., San Diego, CA, USA) following the manufacturer's protocol. Cholesterols were extracted with 200 μL of chloroform: isopropanol: NP-40 (7:11:0.1, v: v: v), and centrifuged at 15,000 × g for 10 min. The extracts were air-dried and then dissolved in 200 μL of assay diluent. Subsequently, 50 μL of the samples or cholesterol standards was added to each well of a 96-well plate containing cholesterol reaction reagent, and the plates were incubated at 37°C for 45 min. Plates were then read with a microplate reader within a wavelength range of 530–570 nm (Li et al., 2017).

### Renal tissue pathology

The kidney tissues of all mice were fixed in 10% phosphate buffered formalin solution, embedded in paraffin, and then sectioned into 2–3 μm thick slices on slides. The slices were stained with periodic acid-Schiff (PAS), and then examined by light microscopy. The degree of glomerulosclerosis was calculated by the percentage of extracellular matrix (ECM) deposition and mesangial expansion and evaluated at 400X power in 20 cortical fields. The mesangial matrix was scored as follows: 1, <10%; 2, 10–25%; 3, 26–50%; 4, 51–75%; 5, 76–95%; 6, >95% (Zhao et al., 2017). The renal sections (8 μm) of fixed frozen mouse kidneys were prepared for Oil Red O and filipin cholesterol staining. For Oil Red O staining, cryosections were air dried for 10 min at room temperature, washed with 60% isopropanol and stained with fresh Oil Red O working solution (Sigma-Aldrich, Saint Louis, MO, USA) for 30 min. After washing with 60% isopropanol three times, the sections were placed under a microscope (Olympus, Tokyo, Japan) to visualize lipid deposition. For filipin cholesterol staining, sections were fixed with 4% paraformaldehyde for 30 min, washed three times with PBS, and then stained with freshly prepared filipin solution (125 μg/mL, Sigma-Aldrich) for 30 min. Next, the slides were washed with PBS, and a drop of glycerol was added. The sections were eventually observed by fluorescence microscopy using an ultraviolet filter set package. All samples were analyzed blindly for overall pathology using the Image-Pro Plus 6.5 software (Media Cybernetics, Bethesda, MD, USA).

### Western blotting analysis

Proteins from the kidney cortex and cultured cells were extracted with radioimmunoprecipitation assay (RIPA) lysis buffer, and analyzed by Western blotting as previously described (Xiao et al., 2012). Primary antibodies against β-actin, LXR (Santa Cruz Biotech., Santa Cruz, CA, USA), PGC-1α and ABCA1 (Abcam, Cambridge, UK) and LI-COR IRDye 800-labeled secondary antibodies (Rockland Immunochemicals, Gilbertsville, PA, USA) were used in this study. Specific signals were detected using the LiCor/Odyssey infrared image system (LI-COR Biosciences, Lincoln, NE, USA) and were quantified using the LiCor/Odyssey followed by analysis with the ImageJ software (NIH).

### RNA extraction and quantitative real-time PCR

Total RNA was extracted from renal tissues and cultured cells and purified using an RNeasy kit according to the manufacturer's instructions (Qiagen, Valencia, CA, USA), and real-time PCR was performed with an Opticon real-time PCR machine (Opticon 2, Bio-Rad Labs., Hercules, CA, USA) using the IQ SYBR Green Supermix reagent (Bio-Rad Labs., Hercules, CA, USA) as previously described (You et al., 2016). The primer sequences used in the present study are listed in Table 1.

**Table 1:** List of primers used for quantitative real-time PCR.

	**Sequence (5′-3′)**
Mus_LXR_forward	CTGCAGGACAAAAAGCTTCC
Mus_LXR_reverse	CCCTTCTCAGTCTGCTCCAC
Mus_ABCA1_forward	CCAGACAGTTGTGGATGTGG
Mus_ABCA1_reverse	GACCTCGCTCTTCCTTCCTT
Mus_PGC-1_forward	CCGAGAATTCATGGAGCAAT
Mus_PGC-1_reverse	TTTCTGTGGGTTTGGTGTGA

### Statistical analyses

All analyses were performed using the GraphPad Prism software version 6.0 (GraphPad Software Inc., La Jolla, CA, USA). The quantitative data were expressed as the mean ± SEM. One-way analysis of variance (ANOVA) was applied for the statistical analysis. *P* < 0.05 was accepted as statistically significant.

## Results

### TSF treatment reduced body weight, UACR, and dyslipidemia in db/db mice

**Figure 1:**
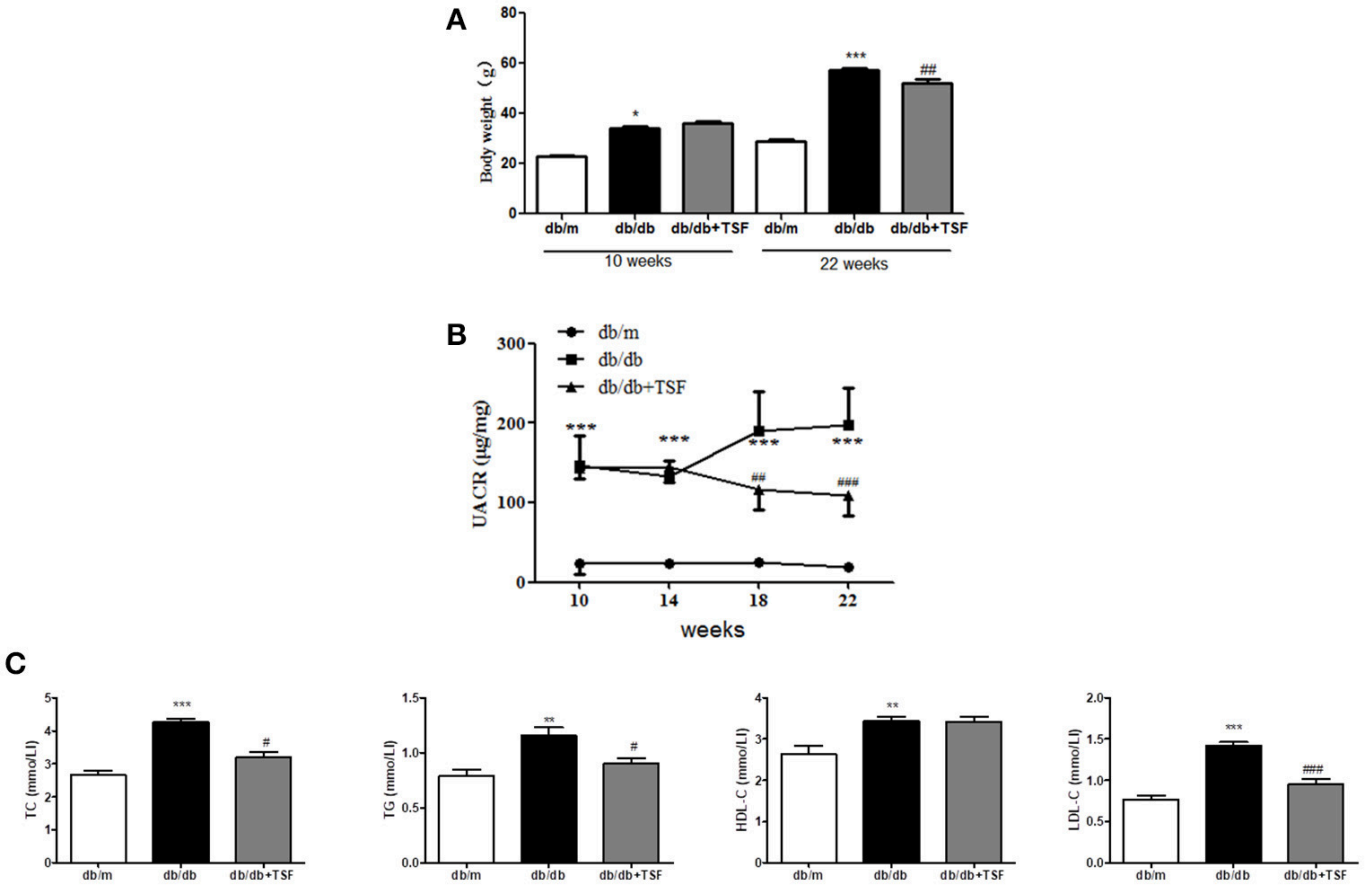
TSF ameliorated renal injuries in db/db mice. Administration of TSF effectively reduced body weight **(A)** and UACR **(B)**. **(C)** TSF reduced dyslipidemia in 22-week-old db/db mice. The data were expressed as the mean ± SEM. ^*^P < 0.05, ^**^P < 0.01, and ^***^P < 0.001 vs. the db/m group; ^#^*P* < 0.05, ^*##*^*P* < 0.01, ^###^*P* < 0.001 vs. the db/db group.

As shown in Figures 1A,B treatment with TSF markedly reduced the body weight and urine albumin-to-creatinine ratio (UACR) in db/db mice.

Compared with db/m mice, serum levels of LDL-C, HDL-C, TC, and TG were significantly increased in db/db mice and were decreased in those treated with TSF, although the level of HDL-C was not significantly affected by the treatment with TSF (Figure 1C).

### Treatment with TSF reduced histological damage and lipid and cholesterol accumulation in the renal tissues of db/db mice

**Figure 2:**
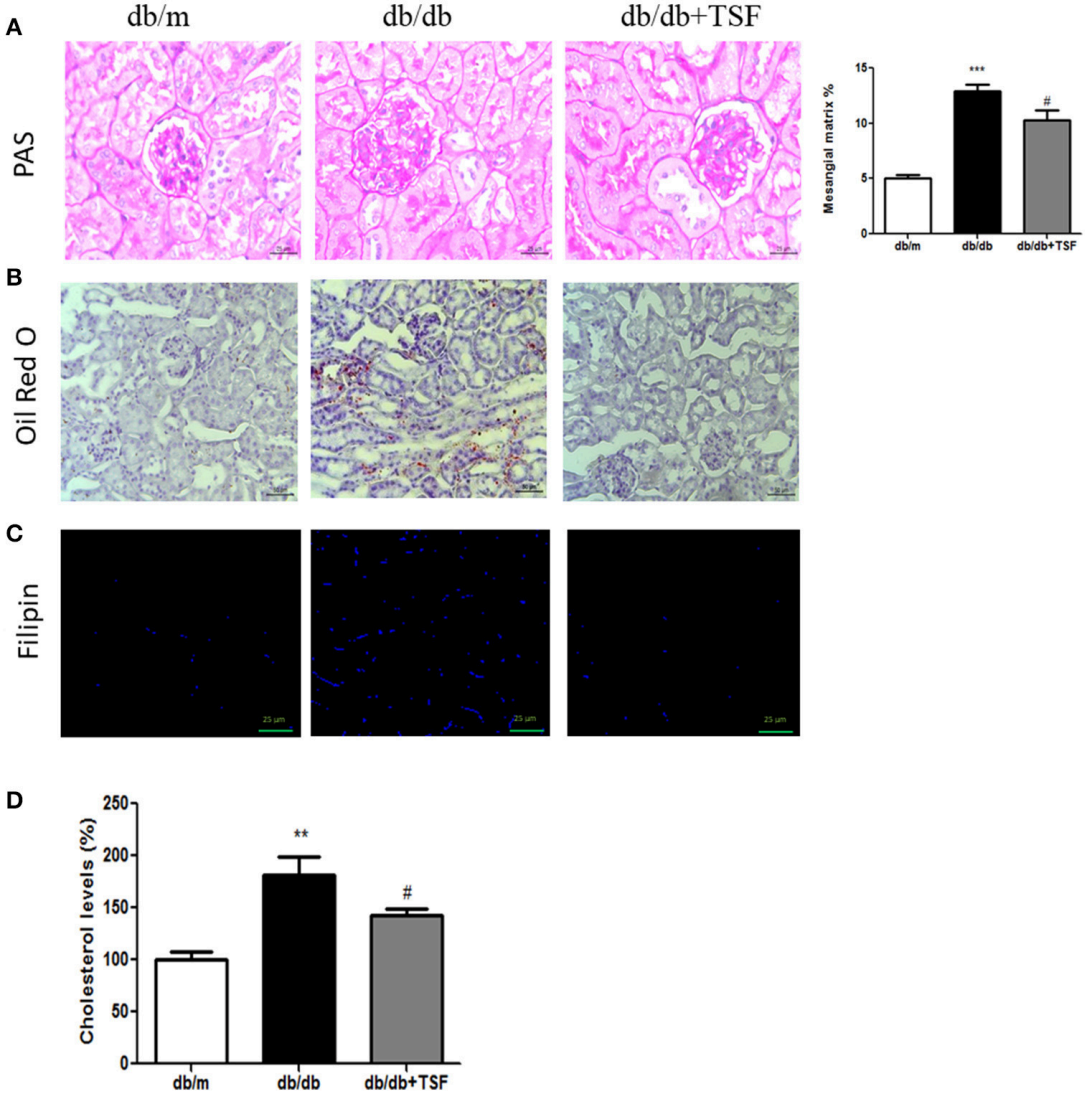
TSF attenuated glomerular mesangial matrix deposition, and lipid and cholesterol accumulation in the renal tissues of db/db mice. **(A)** PAS staining (bar = 25 μm). **(B)** Oil Red O staining (bar = 50 μm). **(C)** Filipin cholesterol staining (bar = 25 μm). **(D)** Analysis with a colorimetric assay demonstrated that TSF decreased total cholesterol levels in the renal tissues of 22-week-old db/db mice. ^**^P < 0.01, ^***^P < 0.001 vs. the db/m group; ^#^*P* < 0.05 vs. the db/db group.

Histological analysis using PAS staining revealed the occurrence of mesangial matrix expansion and extracellular matrix deposition in the kidneys of db/db mice (Figure 2A). The treatment with TSF significantly ameliorated these histological renal injuries in db/db mice (Figures 2A).

Oil Red O and filipin cholesterol staining revealed the occurrence of lipid and cholesterol accumulation in the kidneys of db/db mice, and treatment with TSF significantly prevented the cholesterol accumulation in the kidneys of these mice (Figures 2B,C). Quantitative analysis also revealed that cholesterol levels in the renal cortical tissues were highly increased in db/db mice compared with db/m mice, and TSF treatment decreased the renal cholesterol accumulation in db/db mice (Figure 2D).

### TSF enhanced the expression of PGC-1α, LXR, and ABCA1 in the kidneys of db/db mice

**Figure 3:**
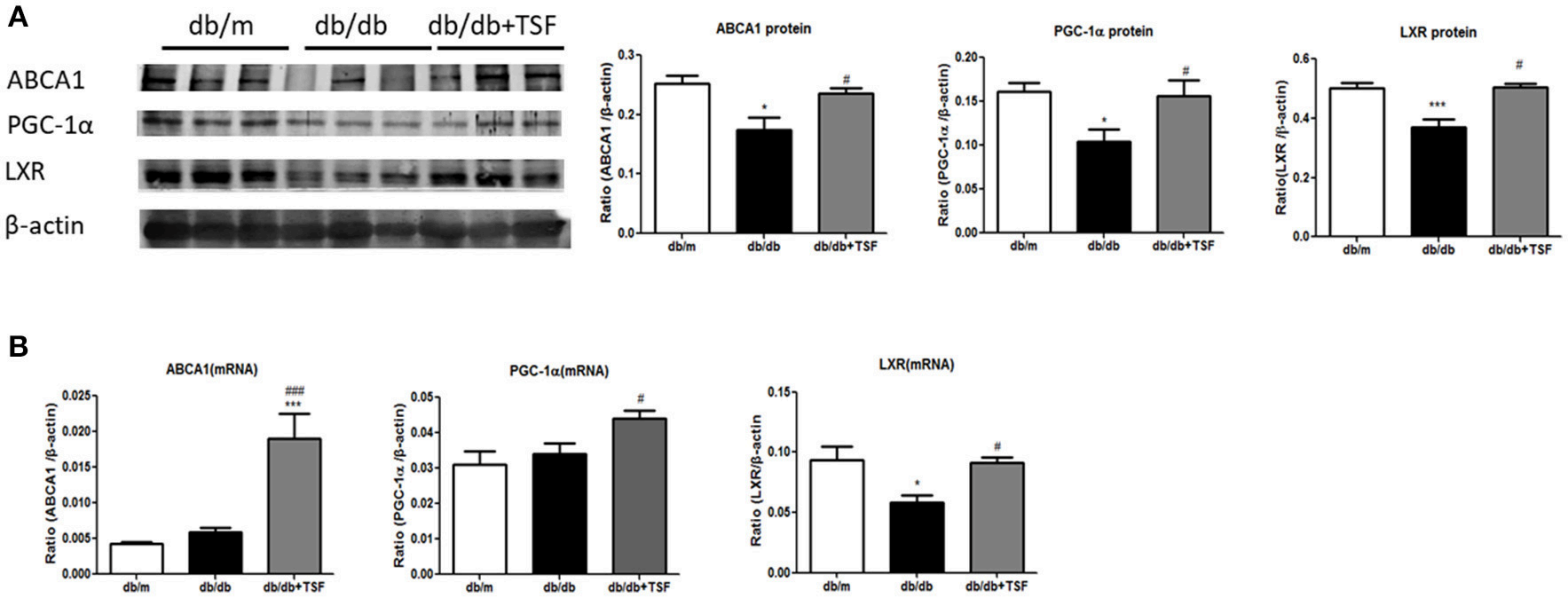
After treatment with TSF, the expression of ABCA1, PGC-1α, and LXR in renal tissues was upregulated in db/db mice. **(A)** Effect of TSF on protein levels of ABCA1, PGC-1α, and LXR by Western blot analysis. **(B)** Effect of TSF on mRNA expression of ABCA1, PGC-1α, and LXR real-time PCR analysis. The data were represented as the mean ± SEM for at least three independent experiments. ^*^P < 0.05 and ^***^P < 0.001 vs. the db/m group; ^#^*P* < 0.05, ^###^*P* < 0.001 vs. the db/db group.

The accumulation of lipid in the glomerular and tubular cells is one of the main features of DN. ABCA1 has been shown to play a key role in promoting renal cholesterol efflux to reduce renal cholesterol accumulation (Perco and Mayer, 2018). We thus investigated whether the PGC-1a-LXR-ABCA1 pathway was involved in the inhibitory effect of TSF on renal cholesterol efflux in db/db mice. Western blot and real-time PCR analysis showed that expression levels of ABCA1, PGC-1α, and LXR were significantly downregulated compared with those in the db/m mice (Figures 3A,B), suggesting the inhibition of renal cholesterol efflux in db/db mice. In contrast, TSF treatment largely increased the expression levels of PGC-1α, LXR, and ABCA1 in db/db mice (Figures 3A,B), suggesting that treatment with TSF might attenuate diabetic renal injury by improving renal cholesterol efflux via the PCG1α-LXR-ABCA1 mechanism.

### TSF promoted renal cholesterol efflux by enhancing the expression of PGC-1α, LXR, and ABCA1 in PA-stimulated mTECs

**Figure 4:**
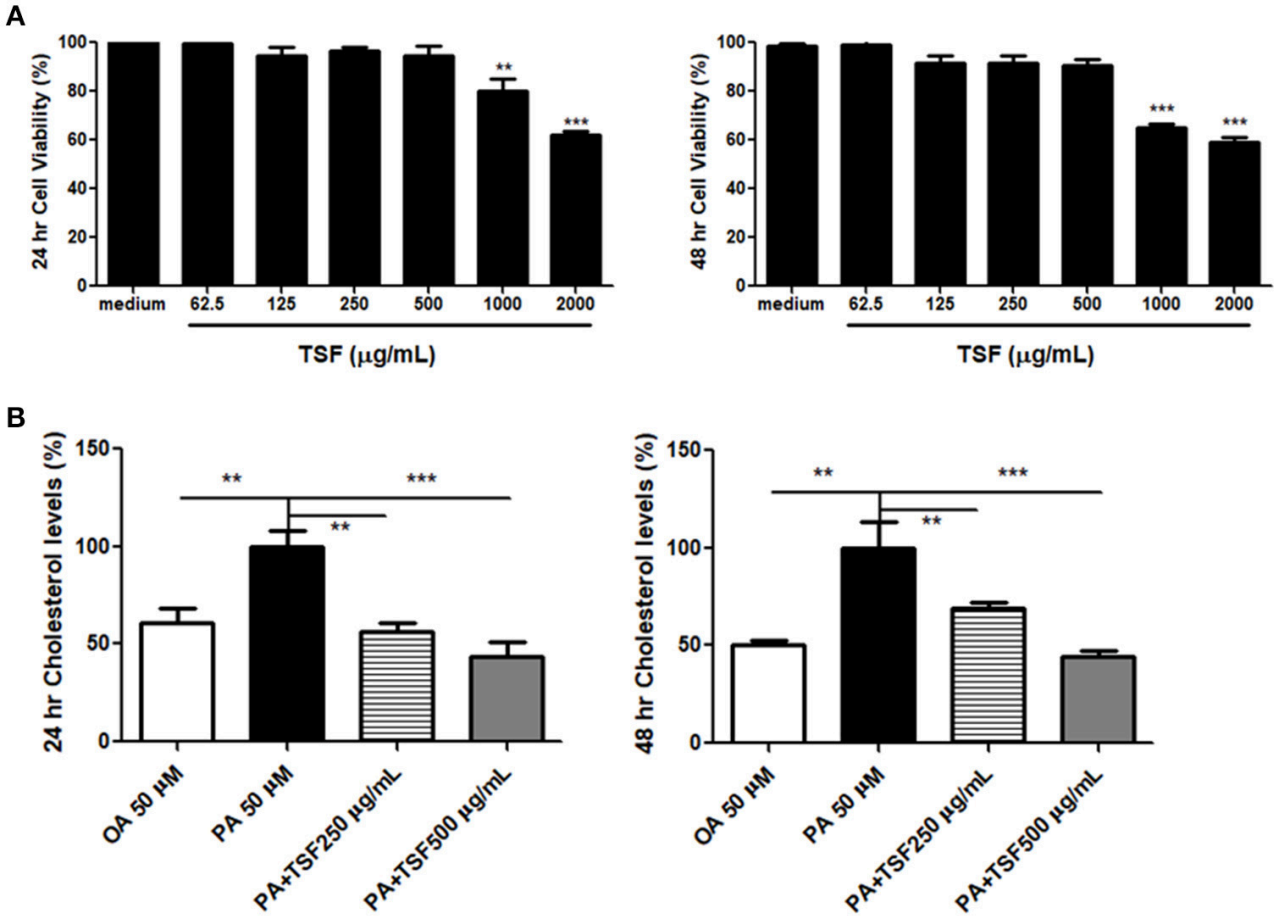
TSF prevented cholesterol accumulation in the mTECs stimulated with PA. **(A)** Dose-dependent effect of TSF on cell viability as determined by the MTT assay for 24 and 48 h. ^**^P < 0.01 and ^***^P < 0.001 vs. the medium group. **(B)** A colorimetric analysis demonstrated that TSF decreased total cholesterol levels in the mTECs stimulated with PA for 24 and 48 h. ^**^P < 0.01 and ^***^P < 0.001 vs. the PA group. The data were represented as the mean ± SEM for at least three independent experiments.

Cholesterol accumulation in renal proximal tubular cells is considered to be a cause of renal lipid accumulation, which plays an important role in the progression of DN (Pedigo et al., 2016). To study the effect of TSF on cholesterol accumulation, mTECs were treated with TSF at various concentrations, ranging from 62.5 to 2,000 μg/mL for 24 and 48 h. The exposure to TSF at concentrations ranging from 62.5 to 500 μg/mL did not result in any significant changes in the survival rate of mTECs (Figure 4A). However, the cytotoxic effect of TSF occurred when the dose was over 1,000 μg/mL (Figure 4A). Based on these results, TSF concentrations <500 μg/mL were used in the subsequent experiments.

The expression of ABCA1 protein was significantly inhibited when induced with PA (50 μM) in mTECs at 48 h (Supplementary Figure S1). Therefore, we used 50 μM of OA and PA to induce mTECs. The colorimetric total cholesterol analysis showed that PA stimulation increased the total cholesterol levels in mTECs, and levels were significantly reduced after TSF treatment (Figure 4B).

**Figure 5:**
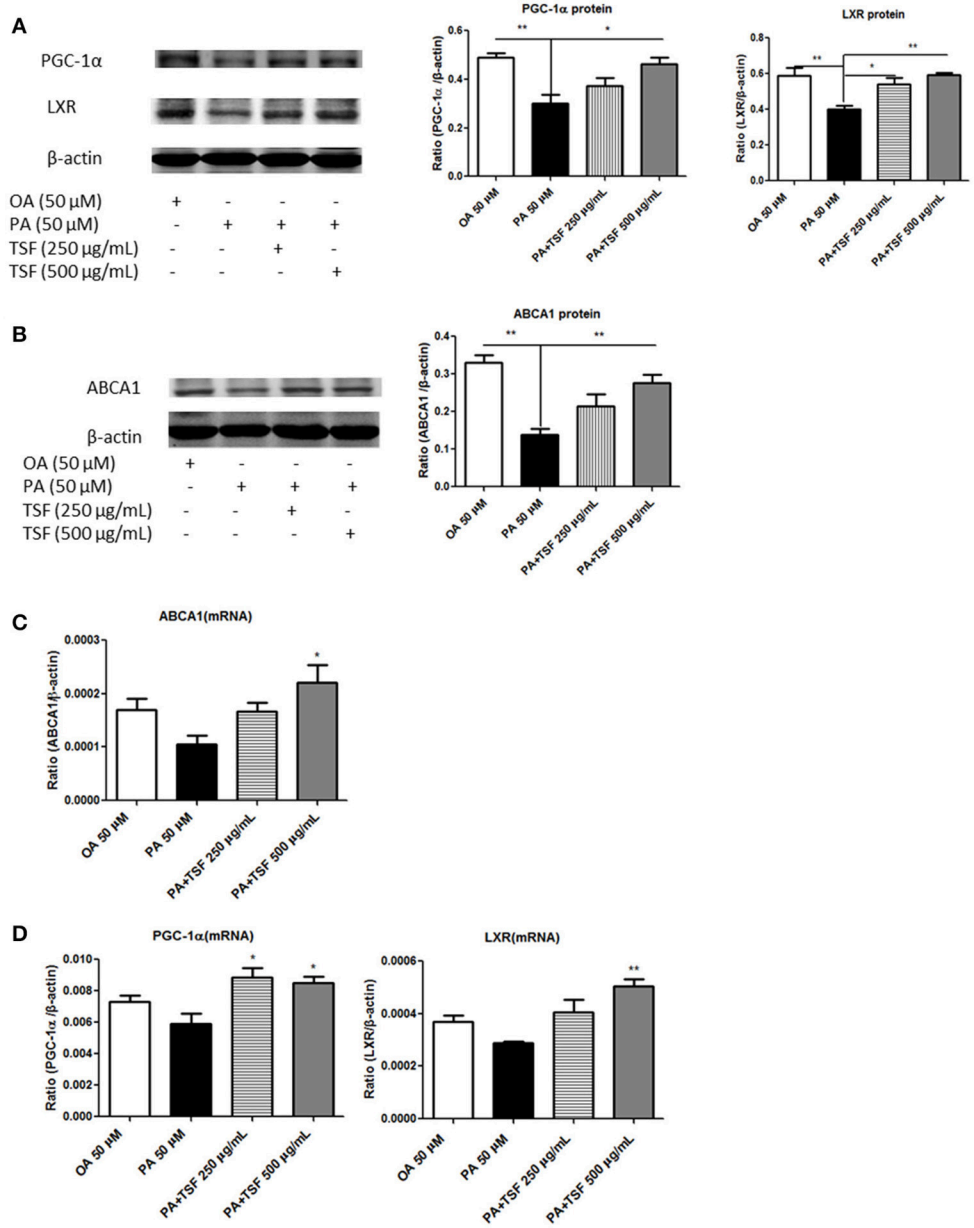
After treatment with TSF, the expression of PGC-1α, LXR, and ABCA1 was upregulated in the mTECs stimulated with PA. **(A)** Western blot analysis of PGC-1α and LXR expression in mTECs cultured for 12 h. **(B)** Western blot analysis of ABCA1 expression in mTECs cultured for 48 h. **(C)** Real-time PCR analysis of PGC-1α and LXR expression in mTEC cells cultured for 3 h. **(D)** Real-time PCR analysis of ABCA1 expression in mTEC cells cultured for 6 h. The data were represented as the mean ± SEM for at least three independent experiments. ^*^P < 0.05, ^**^P < 0.01 vs. the PA group.

We next investigated the mechanisms whereby TSF reduces cholesterol accumulation in PA-stimulated mTECs. Compared with the PA group, the protein expression of PGC-1α was upregulated in mTECs exposed to 500 μg/mL of TSF for 12 h, while the protein expression of LXR was significantly upregulated in mTECs exposed to both 250 and 500 μg/mL of TSF for 12 h. Also, the protein expression of ABCA1 was significantly upregulated in mTECs treated with these two concentrations of TSF for 48 h (Figures 5A,B).

Compared with the PA group, the mRNA expression of LXR was significantly upregulated in mTECs exposed to both concentrations of TSF for 1 h; the mRNA expression of PGC-1α, LXR, and ABCA1 was significantly upregulated in mTECs exposed to TSF 500 μg/mL for 3 h; and the mRNA expression of ABCA1 was upregulated in mTECs exposed to TSF 500 μg/mL for 6 h (Figures 5C,D). Together, these findings demonstrated that TSF positively regulated cellular cholesterol efflux in mTECs.

**Figure 6:**
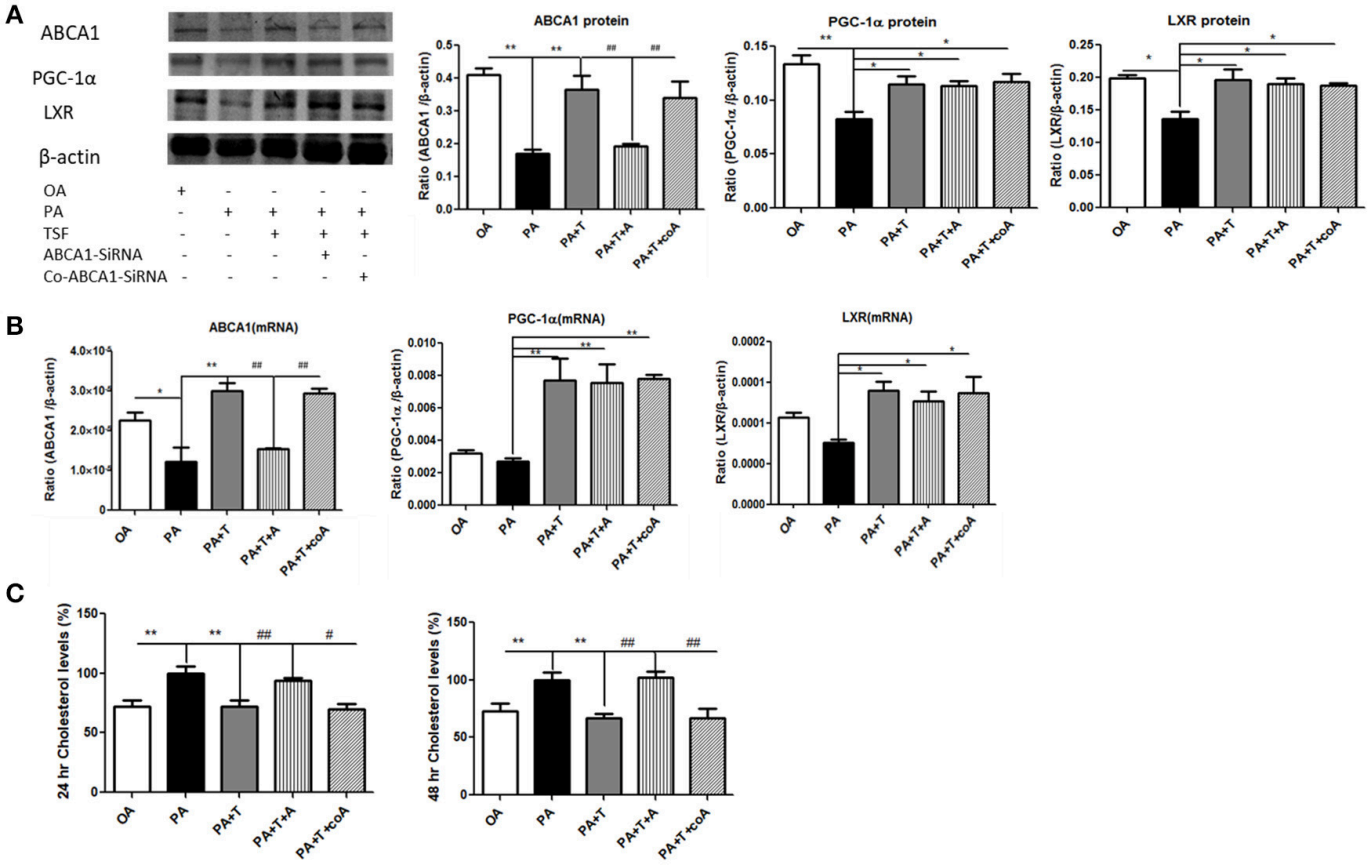
Silencing ABCA1 significantly suppressed the inhibitory effect of TSF on PA-induced total cholesterol levels in mTECs. **(A)** Western blot analysis of ABCA1, PGC-1α, and LXR expression in mTECs cultured for 48 h, and then exposed to OA (oleic acid 50 μM), PA (sodium palmitate 50 μM), PA+T (PA 50 μM+ TSF 500 μg/mL), PA+T+A (PA 50 μM+ TSF 500 μg/mL+ Abca1-SiRNA), or PA+T+A (PA 50 μM+ TSF 500 μg/mL+ Co-Abca1-SiRNA). **(B)** Real-time PCR analysis of ABCA1, PGC-1α, and LXR expression in mTECs cultured for 6 h. **(C)** A colorimetric analysis of total cholesterol levels in the mTECs cultured with Abca1-SiRNA for 24 and 48 h. ^*^P < 0.05, ^**^P < 0.01 vs. the PA group; ^#^*P* < 0.05, ^##^*P* < 0.01 vs. the PA+T+A group.

In order to better understand the functional role of ABCA1 in ABCA1-mediated renal cholesterol efflux, we knocked down ABCA1 in mTECs with the siRNA technique. The results of the colorimetric total cholesterol analysis showed that silencing ABCA1 significantly suppressed the inhibitory effect of TSF on PA-induced total cholesterol levels in mTECs without altering protein and mRNA expression levels of PGC-1α and LXR (Figure 6), revealing that the TSF might act as protection via the ABCA1-dependent mechanism to improve renal cholesterol efflux in DN.

## Discussion

The novel finding of the present study is that renal cholesterol accumulation occurred in the kidney of db/db mice, and it was associated with the suppression of both the PCG-1α-LXR-ABCA1 pathway and renal cholesterol efflux. More significantly, we found that treatment with TSF attenuated diabetic kidney injuries and was associated with improving dyslipidemia and promoting renal cholesterol efflux via the PCG-1α-LXR-ABCA1-dependent mechanism.

Both genetic and environmental factors are linked to the initiation and progression of DN, including genetic pre-disposition, sedentary lifestyle, hypertension, persistent hyperglycemia, and dyslipidemia (Matheus et al., 2013; Ahmad, 2015; Gomes et al., 2015). Dyslipidemia is a known cause of the progression of DN, which is one of the major complications of T2DM (Rutledge et al., 2010). Recently, a growing number of studies have attached importance to renal cholesterol accumulation in DN, and it is regarded as one of the potential mechanisms of lipid metabolism disorder-induced renal injuries (Hao et al., 2012; Toth et al., 2012; Soetikno et al., 2013). In our previous study, we showed that TSF reduced hepatic steatosis via inhibiting lipogenesis and augmenting fatty acid oxidation (Kong et al., 2016). The results of the current study showed that TSF decreased serum triglyceride and cholesterol levels. Importantly, TSF also decreased cholesterol levels in the kidneys.

The efflux of free cholesterol from cells mediated by ABCA1 is the early step in reverse cholesterol transport, and ABCA1 promotes the solubilization of lipids and their release (Hassan et al., 2008). A molecular defect in the ABCA1 gene is the cause of Tangier disease, which leads to premature atherosclerosis, proteinuria, and HDL deficiency (Schaefer et al., 2010). Additionally, ABCA1 genetic variants are strongly associated with the risk of coronary artery disease (Willer et al., 2008). In patients with DN, the increased cholesterol accumulation was associated with ABCA1 downregulation in kidneys (Herman-Edelstein et al., 2014). In type 1 diabetes mellitus and diabetic apolipoprotein E knockout (apoE^−/−^) mice, the expression ABCA1 was downregulated in the kidney (Kruit et al., 2012; Yin et al., 2016). Our *in vivo* data demonstrated that the expression ABCA1 was downregulated in the kidneys of db/db mice, and treatment with TSF significantly decreased renal cholesterol levels and upregulated the expression of ABCA1. Silencing ABCA1 significantly suppressed the inhibitory effect of TSF on PA-induced total cholesterol levels in mTECs. Thus, we hypothesized that the ABCA1-mediated cholesterol efflux might play a major role in renal cholesterol accumulation that occurs in DN.

Additionally, patients with ABCA1 dysfunction and HDL deficiency tend to have chronic low-grade inflammation due to the anti-inflammatory effects of ABCA1 (Birjmohun et al., 2007; Westerterp et al., 2013; Bochem et al., 2015). The decreased ABCA1-mediated cholesterol efflux caused cholesterol-dependent apoptosis in podocytes (Yang et al., 2017b). Therefore, cholesterol accumulation and the downregulation of the expression of ABCA1 in kidneys appear to promote diabetic kidney injury.

Although interventions that increase ABCA1 expression (such as LXR agonists) may be beneficial to DN, they have a relatively high incidence of adverse events (Grefhorst et al., 2002; Katz et al., 2009). Our previous multicenter double-blind randomized placebo-controlled trial and the present study show that treatment with TSF did not produce adverse events (Li et al., 2015). Promotive action of TSF on the upregulation of ABCA1 might contribute to the therapeutic effect in DN.

Renal cholesterol accumulation is due to increased cholesterol synthesis and impairment of cholesterol efflux in glomerular mesangial cells and tubular cells (Tsun et al., 2014). Renal tubular cells represent 90% of the kidney mass and have a high energy demand when free fatty acid oxidation is required (Druilhet et al., 1975; Han et al., 2017). Accordingly, there might be increased danger from the cholesterol accumulation in the renal tubular cells (Yang et al., 2017a). In a previous study, the cholesterol efflux was suppressed and the expression of ABCA1 was downregulated in high glucose-stimulated human glomerular endothelial cells (Yin et al., 2016). The downregulation of ABCA1 expression caused cholesterol-dependent apoptosis in podocytes (Yang et al., 2017b). However, the level of ABCA1 expression in renal tubular cells and its role in renal tubular cell injury remain unclear. Our *in vitro* data demonstrated that TSF treatment significantly decreased cholesterol levels and upregulated the expression of ABCA1 in PA-stimulated mTECs. Additionally, silencing ABCA1 significantly suppressed the inhibitory effect of TSF on PA-stimulated total cholesterol levels in mTECs. These results suggested that TSF might act via the protection of the ABCA1-dependent mechanism to improve renal cholesterol efflux in DN.

Oleic acid (C18:1) is a non-toxic monounsaturated fatty acid, while palmitic acid (C16:0) is the predominant circulating saturated free fatty acid. These acids can enter cells via both free diffusion and protein-mediated transport processes (Druilhet et al., 1975). Both PA and OA can lead to intracellular lipid accumulation in human mesangial cells (Mishra and Simonson, 2005). Moreover, PA can also lead to intracellular lipid accumulation in renal proximal tubular epithelial cells (Abumrad et al., 1998). However, only PA, but not OA, was able to induce apoptosis in mesangial cells and injury to podocytes (Mishra and Simonson, 2005; Lee et al., 2017). Our *in vitro* data demonstrated that the PA-stimulated cholesterol levels in the mTECs were higher than those in mTECs stimulated with OA. Moreover, TSF treatment significantly decreased cholesterol levels in the PA-stimulated mTECs.

There were some limitations in our study. This study focused on the effect of treatment with TSF on renal cholesterol efflux, but the effect of TSF on cholesterol synthesis and uptake was not clear at present. Second, TSF is a CHM containing multiple components. Further studies are required to clarify the mechanism of TSF on regulating cholesterol metabolism in DN.

In conclusion, the present study demonstrated that TSF decreased the cholesterol accumulation in kidneys of db/db mice, and PA stimulated mTECs, through the upregulation of ABCA1. The promotive action of TSF on ABCA1-mediated cholesterol efflux might contribute to the therapeutic effect in DN.

## Author contributions

PeL, HL, and PiL: Designed research; PeL, LP, PT, and XH: Performed experiments; HjZ, TZ, MY, and HlZ: Contributed to animal experiments; PeL and LP: Analyzed data; PeL and PiL: Drafted the manuscript.

### Conflict of interest statement

The authors declare that the research was conducted in the absence of any commercial or financial relationships that could be construed as a potential conflict of interest.

## Supplementary material

Supplementary Figure S1 **(A)** Western blot analysis of ABCA1 expression in mTEC cells induced with different concentrations of PA cultured for 48 h. ^*^*P* < 0.05 and ^**^*P* < 0.01, and ^***^*P* < 0.001 vs. the OA 50 μM group; **(B)** Western blot analysis of ABCA1 expression in mTECs cultured for 24 h. ^*^*P* < 0.05 and ^**^*P* < 0.01, vs. the PA group. **(C)** Western blot analysis of ABCA1 expression in the mTECs cultured with ABCA1-SiRNA for 24 h. ^*^*P* < 0.05 and ^**^*P* < 0.01, vs. the PA group; ^#^*P* < 0.05 vs. the PA+T+A group.

## Abbreviations

ABC: ATP-binding cassette
ABCA1: ATP-binding cassette subfamily A member 1
ANOVA: One-way analysis of variance
apoA1: apolipoprotein A1
CHM: Chinese herbal medicine
DN: diabetic nephropathy
DMEM: dulbecco's modified eagle medium
ECM: extracellular matrix
eGFR: estimated glomerular filtration rate
ESRD: end-stage renal disease
FBS: fetal bovine serum
HDL-C: high-density lipid cholesterol
HPLC: high performance liquid chromatography
LDL-C: low-density lipid cholesterol
LXR: Liver X receptors
mTECs: mouse tubular epithelial cells
OA: oleic acid
PA: sodium palmitate
PAS: periodic acid-Schiff
PBS: phosphate buffer saline
PGC-1α: peroxisome proliferator-activated receptor γ coactivator 1-α
RIPA: radioimmunoprecipitation assay
TC: total cholesterol
TG: triglycerides
TSF: Tangshen Formula
T2DM: type 2 diabetes mellitus
UACR: urine albumin-to-creatinine ratio.
