# Supplementary figures and images for: Tangshen Formula Attenuates Diabetic Nephropathy by Promoting ABCA1-Mediated Renal Cholesterol Efflux in db/db Mice
Source: Front Physiol. 2018 Apr 6;9:343. doi: 10.3389/fphys.2018.00343 (PMC5897509; doi:10.3389/fphys.2018.00343)

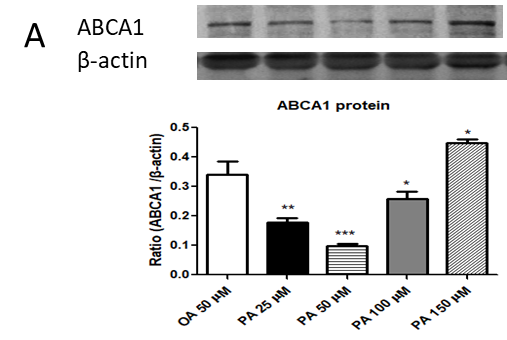

Supplement: Supplementary Figure S1 — (A) Western blot analysis of ABCA1 expression in mTEC cells induced with different concentrations of PA cultured for 48 h. *P < 0.05 and **P < 0.01, and ***P < 0.001 vs. the OA 50 μM group; (B) Western blot analysis of ABCA1 expression in mTECs cultured for 24 h. *P < 0.05 and **P < 0.01, vs. the PA group. (C) Western blot analysis of ABCA1 expression in the mTECs cultured with ABCA1-SiRNA for 24 h. *P < 0.05 and **P < 0.01, vs. the PA group; #P < 0.05 vs. the PA+T+A group. [file Image1.TIF]

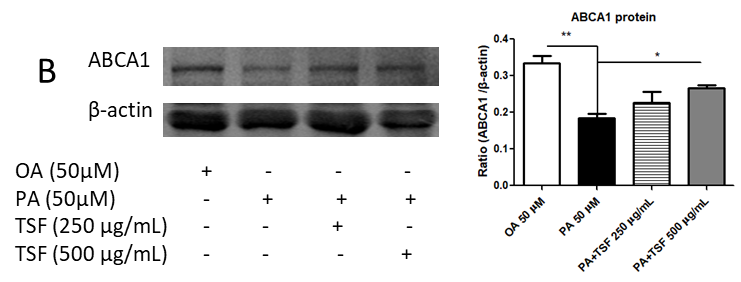

Supplement: Supplementary file 2 [file Image2.TIF]

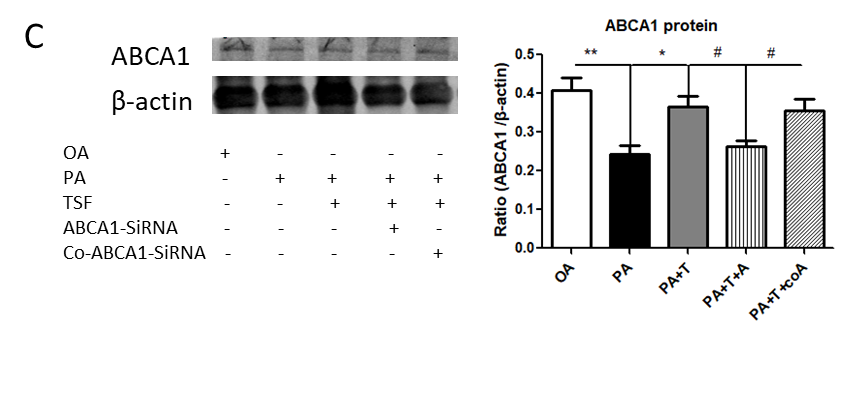

Supplement: Supplementary file 3 [file Image3.TIF]
